# Lifelong Impact of Prenatal Stress: Exacerbated Memory Impairments and Gene Expression Changes Under Adult Chronic Stress

**DOI:** 10.1002/brb3.71073

**Published:** 2025-11-28

**Authors:** Manijeh Dogani, Nayere Askari, Mohammad‐Reza Vaez‐Mahdavi

**Affiliations:** ^1^ Department of Biology Faculty of Science Shahid Bahonar University of Kerman Kerman Iran; ^2^ Faculty of Basic Sciences Shahed University Tehran Iran; ^3^ Immunoregulation Research Center Shahed University Tehran Iran; ^4^ Health Equity Research Center Shahed University Tehran Iran

**Keywords:** prenatal stress, learning, memory, chronic unpredictable stress, social instability stress, glutamate receptors, TLR2/4, BDNF

## Abstract

**Introduction:**

Chronic stress can have significant impacts on both physical and mental health, including affecting learning and memory processes. Research has shown that exposure to stress during prenatal and neonatal stages can have long‐lasting effects on individuals. The objective of this study is to investigate how prenatal stress influences an organism's response to adult stress in terms of memory and learning processes. This comprehensive approach aims to examine the impact of prenatal and adult stress on cognitive functions and gene expression in specific brain regions.

**Methods:**

The study involved subjecting pregnant female rats to restraint stress, followed by exposing male offspring to chronic unpredictable stress (CUS) and social instability stress in adulthood for 3 weeks. The researchers then assessed passive avoidance, active avoidance, and spatial learning and memory using the shuttle box and Morris Water Maze tasks, respectively. Additionally, the expression levels of glutamate receptors, brain‐derived neurotrophic factor (BDNF), tropomyosin receptor kinase B (TrkB), and Toll‐like receptors (TLR2/4) genes in the rats' hippocampus and prefrontal cortex were analyzed.

**Results:**

According to our data, exposure to prenatal maternal stress led to impairments in learning and memory. These disorders are exacerbated in prenatally stressed rats exposed to adulthood stress. TLR2 and TLR4 mRNA levels were significantly elevated. In contrast, the expression of BDNF and TrkB and NMDA and AMPA receptor genes decreased compared to the control.

**Conclusion:**

Collectively, these results indicate that exposure to stressors during the prenatal period is associated with potential long‐term impairments in memory formation and/or retrieval, which may persist into adulthood and be exacerbated by chronic stress experienced later in life.

## Graphical abstract

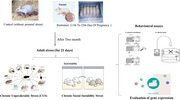

**Abbreviations**
AMPA: alpha‐amino‐3‐hydroxy‐5‐methyl‐4‐isooxazole‐propionic acid.
BDNF: brain‐derived neurotrophic factor.
GAPDH: Glyceraldehyde 3‐phosphate dehydrogenase
NMDA: N‐methyl‐D‐aspartate receptor.
TLR2: Toll‐like receptors 2.
TLR4: Toll‐like receptors 4.
TrkB: tropomyosin receptor kinase B.

## 1 Introduction

The fetal period is one of the most sensitive and important periods in an individual's life. The interaction between genetics and environmental factors during the embryonic period determines the developmental course of the offspring's growth and mental health (Schlotz and Phillips 2009). The stress experienced by the mother during pregnancy, especially in the last third of pregnancy, which is the critical period for the organization of neuronal synapses, can have harmful effects on the speed of development and physical and mental health of the fetus (Malave et al. 2022; Weinstock 2005). Accumulating research indicates that prenatal exposure to maternal stress increases the risk of behavioral and mental health issues later in life (Van den Bergh et al. 2020). Research indicates that the most common form of stress experienced by individuals comes from their social context (called “social stress”) and is often perceived as more intense than other types of stressors (Boltivets et al. 2024; Linares et al. 2020). Experiencing chronic stress can lead to emotional, behavioral, and physiological changes, thereby increasing the risk of developing both mental and physical illnesses (Patel et al. 2019).

The hippocampus and prefrontal cortex are two important brain regions involved in learning and memory formation. Solid evidence shows that stress can cause stable changes in these areas of the brain (McEwen 2022; McEwen et al. 2016). It affects memory formation by affecting neurotransmitters, hormones, and numerous peptides that are released in response to stressful events (Calvo and Gutierrez‐Garcia 2016; de Quervain et al. 2019; Hosseini et al. 2024). It has been indicated that the chronic elevation of maternal glucocorticoids crosses the placental barrier, disrupting hippocampal neurogenesis and synaptic plasticity, which in turn impairs offspring performance in spatial and avoidance learning tasks (McGowan and Matthews 2018).

At the cellular and molecular level, stress exerts its effects on memory decline by changing the expression of several genes, including glutamate receptors, brain‐derived neurotrophic factor (BDNF), tropomyosin receptor kinase B (TrkB), and Toll‐like receptors 2 and 4 (TLR2/4) (Furuyashiki and Kitaoka 2019; Mohseni‐Moghaddam et al. 2022; Yuen et al. 2009). Glutamate is one of the most important excitatory nerve mediators in the brain, so the function of all excitatory synapses in the brain is closely related to glutamatergic synapses. The effects of glutamate are mediated by a combination of different glutamate receptors, which are classified into ionotropic and metabotropic receptors (Hassel and Dingledine 2012). The role of inotropic receptors, AMPA and NMDA, has been proven in cognitive processes, such as memory, learning, and attention. Also, BDNF and its receptor, tyrosine kinase b, are necessary for synaptic flexibility as well as learning and memory dependent on the hippocampus. Stress affects the expression of BDNF or changes in the expression of tyrosine kinase receptors, disrupting memory formation and neuron degeneration (Finsterwald and Alberini 2014; Gulpinar and Yegen 2004). TrkB is the high‐affinity receptor for BDNF, mediating neurotrophic signaling that underlies neuronal survival, synaptic plasticity, and cognitive function (Gupta et al. 2013).

In addition, TLRs are a group of membrane‐bound receptor proteins that participate in response to infection, stress, and injury by initiating an innate immune response and activating inflammatory signalling pathways. TLR‐s cause the release of various inflammatory cytokines through the activation and signalling of the nuclear factor, such as NF‐κB (Mohanty et al. 2019; Zhao et al. 2021). Moreover, these receptors are expressed in many different types of neural cells and have been linked to the modulation of cognitive function as well as neural plasticity in the brain under physiological conditions (Chen et al. 2019). It has been found that the increase in the expression of TLR4, under the influence of stress, causes disorders in spatial memory and hippocampal neurogenesis in adults through the reduction of proliferation and differentiation of neurons (Connolly et al. 2021).

Although the detrimental impact of prenatal stress alone is well characterized, far less is known about how early‐life adversity interacts with stressors encountered in adulthood to shape cognitive outcomes. This study was designed to fill a critical gap in our understanding of how prenatal adversity primes the brain for later‐life challenges by examining the combined effects of maternal restraint stress and adult chronic unpredictable plus social instability stress on learning, memory, and associated molecular pathways. We evaluated cognitive performance in male offspring of stressed dams while quantifying the expression of glutamate receptor subunits (NMDA and AMPA), BDNF, TrkB, and TLR2/4 genes in the animals' hippocampus and prefrontal cortex. We hypothesize that prenatal stress not only impairs baseline cognitive function but also exacerbates the deleterious impact of adult stress through downregulation of neurotrophic signaling and upregulation of innate immune receptors. By integrating behavioral and gene‐expression endpoints, our comprehensive approach aims to elucidate the mechanistic underpinnings of stress‐induced memory deficits and identify potential molecular targets for intervention.

## 2 Materials and Methods

### 2.1 Animals

This experimental study was conducted using 20 female and 10 male Wistar rats. At the first step, two females were mated with one male, after 12 h, the vaginal smear was checked and the pregnancy was confirmed by presence of sperm, considered “day 0” of pregnancy. At the next step, the males were separated, and the pregnant females were divided into two groups: maternal stress (MS; *N* = 10) and control (C; *N* = 10). Following parturition, each dam and her litter were housed individually until postnatal day 28 to standardize maternal care. At weaning, male offspring were regrouped into cages of four. A total of 48 male pups were included in the study. When they reached two months of age, with body weights ranging from 170–190 grams, these adult males were used for experimental procedures. The rats were randomly divided into six groups, with eight animals in each group. They were housed in the same location under a 12‐h light/dark cycle, with the temperature maintained at 22 ± 2°C. Food and water were provided ad libitum. This study protocol was approved by the ethical committee of Shahed University with the registry code IR.SHAHED.REC.1397.046.

### 2.2 Experimental Design

The male rats were randomly divided into the following six groups (eight rats per group).

1. Control group.
2. Prenatal maternal stress (MS) group.
3. Chronic Unpredictable Stress (CUS) group.
4. Chronic Social Instability Stress (Instability) group.
5. Prenatal maternal stress + Chronic Unpredictable Stress (MS + CUS) group.
6. Prenatal maternal stress + Chronic Social Instability Stress (MS + instability) group.

#### 2.2.1 Prenatal Maternal Stress Protocol

After identifying the first day of pregnancy through the formation of vaginal plaque, from the eleventh day of gestation until delivery on day 22, pregnant rats were placed in a restrainer three times daily (at 9:00 a.m., 12:00 p.m., and 5:00 p.m.). Each session lasted 45 min.

#### 2.2.2 Chronic Unpredictable Stress (CUS) Protocol

Over a period of 21 days, rats were subjected to a variety of stressors, encountering two distinct types each day. To minimize habituation, the sequence of stressors was varied on a weekly basis. Details of the CUS protocol implemented in this study are presented in Table 1.

**TABLE 1:** 21 days Protocol for chronic stress induction.

Day	1	2	3	4	5	6	7	8
**Stressor**	15 min forced swim (20°C), 1 min tail pinch	12 h cage tilting (45°C), 1 h cage rotation	Reversal of the light/dark cycle, 1 h cold room (4°C)	12 h wet bedding, crowded Cage	24 h food deprivation, 1 h restraint	12 h cage tilting (45°C), crowded cage	24 h water deprivation, 1 h cold room isolation	Reversal of the light/dark cycle, 1 min tail pinch
**Day**	**9**	**10**	**11**	**12**	**13**	**14**	**15**	**16**
**Stressor**	Cold room (4°C), 1 h cage rotation	24 h water and food deprivation, 12 h cage tilting(45°C)	15 min forced swim (20°C), 1 h restraint	Reversal of the light/dark cycle, 24 h food deprivation	1 min tail pinch, cold room (4°C)	24 h water deprivation, 1 h restraint	12 h wet bedding, 12 h cage tilting (45°C)	1 h cage rotation, reversal of the light/dark cycle
**Day**	**17**	**18**	**19**	**20**	**21**			
**Stressor**	1 h restraint, crowded cage	12 h wet bedding, 1 min tail pinch	Reversal of the light/dark cycle, 12 h cage tilting (45°C)	15 min forced swim (20°C), 24 h water Deprivation	1 h cage rotation, crowded cage			

#### 2.2.3 Chronic Social Instability Stress

The composition of the cages was altered every three days over a period of 3 weeks. During each rotation, three mice from different cages were placed together in a clean cage, with the rotation schedule randomized (Hosseini et al. 2023; Omidi et al. 2020).

### 2.3 Behavioural Assays

#### 2.3.1 Morris Water Maze (MWM)

Spatial learning and memory were measured based on the MWM test, which included two stages (acquisition and probe tests) that were performed in one day. The acquisition test consists of 4 consecutive blocks with four trials in each block. In each block, rats had 60 s to find the hidden platform in each trial. When the mouse finds the platform, it stays on the platform for 30 s. If the platform is not found, it is slowly guided to the platform for 30 s. Total escape latency, distance travelled, and swimming speed were recorded. 2 h after the acquisition test, a probe test was performed. At this point, the hidden platform was removed from the pool, and the rats swam for 60 s. The time and distance spent in the target quadrant were recorded and analyzed as a measure of maintaining spatial memory (Morris 2008).

#### 2.3.2 Shuttle Box Test (Passive Avoidance Learning)

The apparatus consisted of separate light and dark chambers with a grid floor. A polymethyl methacrylate (Plexiglass) gate separated the two compartments. For the habituation trial, the rats were placed in the light chamber for 30 min before the acquisition trial, allowing them to freely enter the dark chamber. During the acquisition phase, each rat was individually placed in the light chamber, where it remained for 5 s before the guillotine gate was opened. Once the rat entered the dark chamber, the guillotine door was closed, and a constant electric shock (0.5 mA, 50 Hz, lasting 4 s) was delivered through the grid floor. After 30 s, the rats were returned to their home cage, and 5 min later, the same procedure was repeated. If the rats did not enter the dark chamber within 300 s, passive avoidance learning was considered successful; otherwise, they received the shock again. The number of acquisition sessions was recorded. Twenty‐four hours later, during the retention test, the rats were again placed in the light chamber and allowed to cross into the dark chamber. Step‐through latency (STL) was recorded, with a maximum duration of 300 s.

### 2.4 Evaluation of Gene Expression (real‐time PCR)

At the end of the treatment days, the rats were anesthetized, and after scarifying, their hippocampal and prefrontal tissue were harvested for RNA extraction. All the extraction steps was performed by the manual protocol of the Cinna Gen Co with the RNX‐Plus reagent, and the pellet was dissolved in 30 µL of DEPC‐treated water. The obtained samples were stored in a freezer at ‐80°C to prevent quality loss. Then, cDNA was synthesized by the Easy cDNA Synthesis Kit (pars Tous) according to the company's instructions. To investigate gene expression, a Rotro gene detection system and Real Q Plus 2× Master Mix were employed. The real‐time PCR protocol for assessing gene expression included an initial denaturation step at 95°C for 10 min, followed by 40 cycles of PCR. Each cycle consisted of a denaturation phase at 95°C for 20 s, an annealing phase at 60°C for 30 s, and an elongation phase at 72°C for 30 s. Primer sequences, RT‐PCR product lengths, and NCBI accession numbers are detailed in Table 2. All samples were evaluated in duplicate, and the means of these duplicates were utilized for subsequent analyses. The linearity and efficiency of PCR amplification were assessed using standard curves generated from varying amounts of cDNA. Relative mRNA levels were calculated using the expression 2^−ΔΔCT^.

**TABLE 2:** Primer sequences, RT‐PCR fragment lengths, and NCBI accession numbers.

Primer name	Primer sequence	Size of PCR product	NCBI accession number
NMDA (*Grin1*)	F: CTCATCTCTAGCCAGGTCTACG R: GTCAGAGTAGATGGACATTCGGG	147	NM_001287423.1
AMPA (*Gria1*)	F: GGACAACTCAAGCGTCCAGA R: CCACACAGTAGCCCTCATAGC	125	NM_031608.2
TLR4	F: CGGAAAGTTATTGTGGTGGTGT R: GGACAATGAAGATGATGCCAGA	173	NM‐021578.2
TLR2	F: GGGTTCTGACATTGGAGTCC R: CAGTGTCCTGTAAGGATTTCC	182	XM_008761102/1
BDNF	F: CGTGATCGAGGAGCTGTTGG R: CTGCTTCAGTTGGCCTTTCG	343	XM_008762078
TRK‐B	F: TGACGCAGTCGCAGATGCTG R: TTTCCTGTACATGATGCTCTCTGG	245	NM_012731
GAPDH	F: GTCTTCACCACCACGGAGAAGGC R: ATGCCAGTGAGCTTCCCGTTCAGC	392	NM_017008

### 2.5 Statistical Analysis

The results were assessed for normality and subsequently analyzed using a one‐way ANOVA method followed by Tukey's HSD post hoc test, employing IBM SPSS Statistics (version 19). The findings are presented as mean values ± SEM to evaluate statistical differences among the various groups, with a *p*‐value of < 0.05 deemed statistically significant.

## 3 Result

### 3.1 MWM Test

#### 3.1.1 Acquisition Test

The results of the MWM test indicated that exposure to maternal and adult stress, specifically the CUS and INS groups, led to a significant increase in the time and distance required to locate the concealed platform, as compared to the control group. Maternal stress experienced in the MS + CUS and MS + Instability groups resulted in a respective increase in total duration (from 17.4 s to 23.8 s and 28.5 s) and total distance traveled (from 2.5 m to 4.2 m and 3.6 m).

**FIGURE 1:**
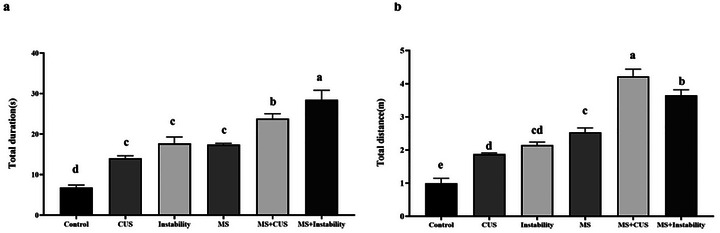
Illustrates the impact of maternal and social stress on spatial learning deficits. Each point represents the mean ± SEM. maternal stress (MS) group, social instability stress (Instability) group, and chronic unpredictable stress (CUS) group. Groups that differ significantly are indicated with a different letter (*p* < 0.05).

Furthermore, the findings revealed that initial exposure to stress in the MS + CUS and MS + Instability groups led to a greater increase in time and total duration compared to the CUS and Instability groups alone. In the CUS groups, the time required increased from 14 s to 23.8 s, and the total distance increased from 2.1 m to 4.2 m. Similarly, in the instability groups, the time increased from 17.6 s to 28.5 s and the total distance increased from 2.5 m to 3.6 m (Figure 1).

#### 3.1.2 Probe Test

**FIGURE 2:**
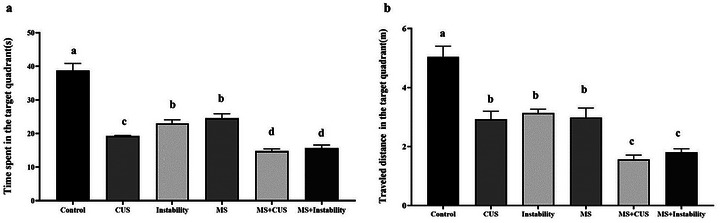
Illustrates the impact of various stressors on memory deficits. Each point represents the mean ± SEM. Maternal stress (MS) group, social instability stress (Instability) group, and chronic unpredictable stress (CUS) group. Groups that differ significantly are indicated with a different letter (*p* < 0.05).

The results revealed that maternal stress and adult stress, as observed in the CUS and INS groups, had a significant impact on the distance traveled and time spent in the target area, in comparison to the control group. In the MS + CUS and MS + Instability groups, the time spent in the target area decreased by 10 s and 9 s, respectively, when compared to the maternal stress group. Furthermore, the traveled distance decreased by 1.4 m and 1.1 m, respectively, when compared to the maternal stress group (Figure 2).

In summary, the results demonstrated that maternal stress, specifically in the MS + CUS and MS + Instability groups, resulted in a decrease in both the time and distance spent in the target area when compared to the CUS and Instability groups. In the CUS groups, the time spent decreased from 19.5 s to 14.2 s, while the traveled distance decreased from 2.92 m to 1.58 m. Similarly, in the instability groups, the time spent decreased from 22.9 s to 15.02 s, and the total distance traveled decreased from 3.13 m to 1.8 m.

### 3.2 Passive Avoidance Learning Test

**FIGURE 3:**
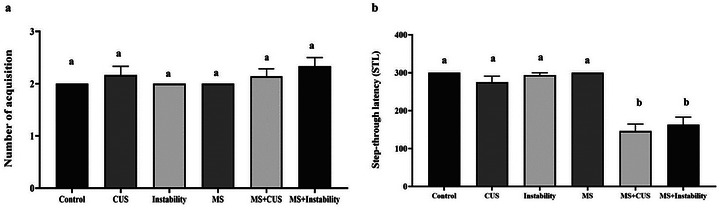
Effect of different stresses on spatial learning and memory deficits in the passive avoidance test. Each point represents the mean ± SEM. Maternal stress (MS) group, social instability stress (Instability) group, and chronic unpredictable stress (CUS) group. Groups that differ significantly are indicated with a different letter (*p* < 0.05).

The results of the passive avoidance test indicated that there was no statistically significant difference in the number of acquisition trials for successful learning among the groups under study. As shown in Figure 3, maternal stress, CUS, and instability did not lead to a significant change compared to the control group. Moreover, there was no difference in the amount of STL between the maternal stress and CUS + MS groups, nor between the instability and MS groups. However, both the CUS + MS and instability + MS groups exhibited a significant decrease in STL time compared to the control and MS groups. Additionally, the results showed that the STL time in the CUS groups decreased from 275 s to 146 s, while in the instability groups, it decreased from 293 s to 163 s.

### 3.3 Gene Expression Analysis

#### 3.3.1 BDNF and TrkB Gene Expression

**FIGURE 4:**
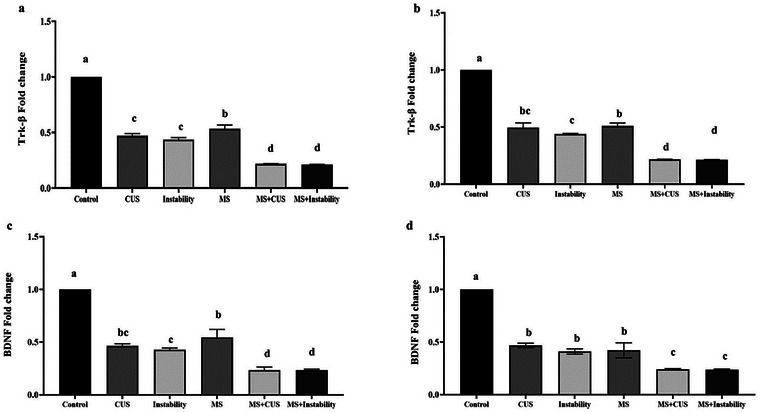
Effect of different stresses on the gene expression of BDNF and TrkB in the hippocampal tissue (a and c) and the prefrontal cortex (b and d). Each point represents the mean ± SEM. Maternal stress (MS) group, social instability stress (instability) group, chronic unpredictable stress (CUS) group. Groups that differ significantly are indicated with a different letter (*p* < 0.05).

Figure 4 illustrates a significant decrease in the expression of BDNF and TrkB in hippocampal and prefrontal tissues in the MS, CUS, and instability groups. The expression levels of these genes in these groups were reduced by approximately 50% compared to the control group. Furthermore, the results indicate that both MS + CUS and MS + Instability stress conditions resulted in a statistically significant reduction in BDNF and TrkB mRNA expression compared to the MS group. Maternal stress also caused a substantial exponential decrease in the expression of these two genes in the CUS + MS and Instability + MS groups, in comparison to the CUS and Instability groups.

#### 3.3.2 TLR2 and TLR4 Gene Expression

**FIGURE 5:**
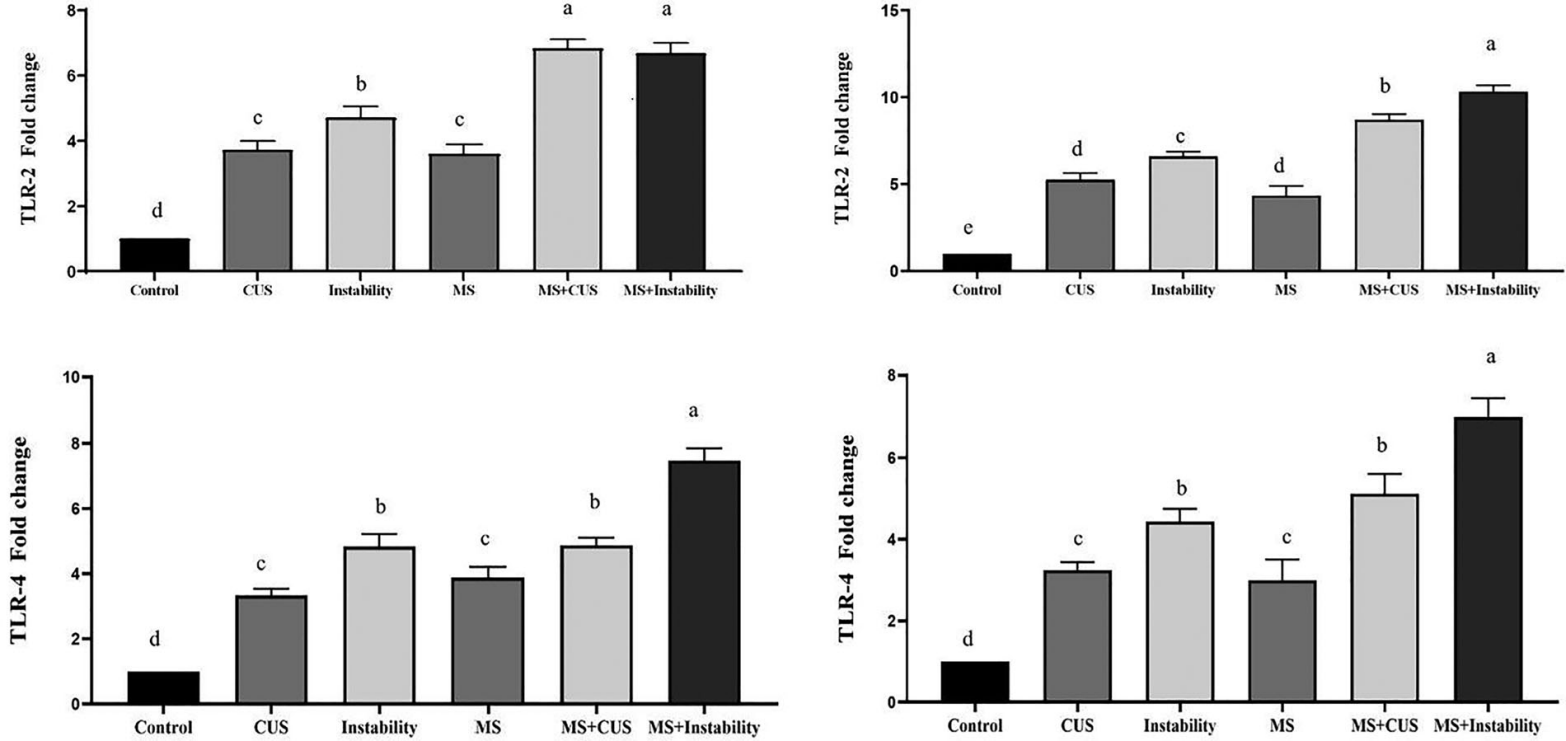
Effect of different stresses on the gene expression of TLR2 and TLR4 in the hippocampal tissue (a and c) and the prefrontal cortex (b and d). Each point represents the mean ± SEM. Maternal stress (MS) group, social instability stress (Instability) group, and chronic unpredictable stress (CUS) group. Groups that differ significantly are indicated with a different letter (*p* < 0.05).

As depicted in Figure 5, the findings indicate a significant elevation in TLR2 and TLR4 mRNA levels in the prefrontal and hippocampal tissues of animals in the maternal stress, CUS, and Instability groups when compared to the control group. In the maternal stress group, the expression level of these genes was approximately three to four times higher than that in the control group. Similarly, in the CUS group, the expression level of these two genes was three to four times higher than in the control group, while in the instability group, it increased up to five times. Our data demonstrates that rats subjected to MS + CUS and MS + Instability exhibited a substantial increase in mRNA expression of TLR2 and TLR4 compared to the maternal stress group, almost doubling it. Furthermore, in the CUS + MS and Instability + MS groups, the expression level of both genes increased by 2–3 units compared to the CUS and Instability groups.

#### 3.3.3 AMPA and NMDA Gene Expression

**FIGURE 6:**
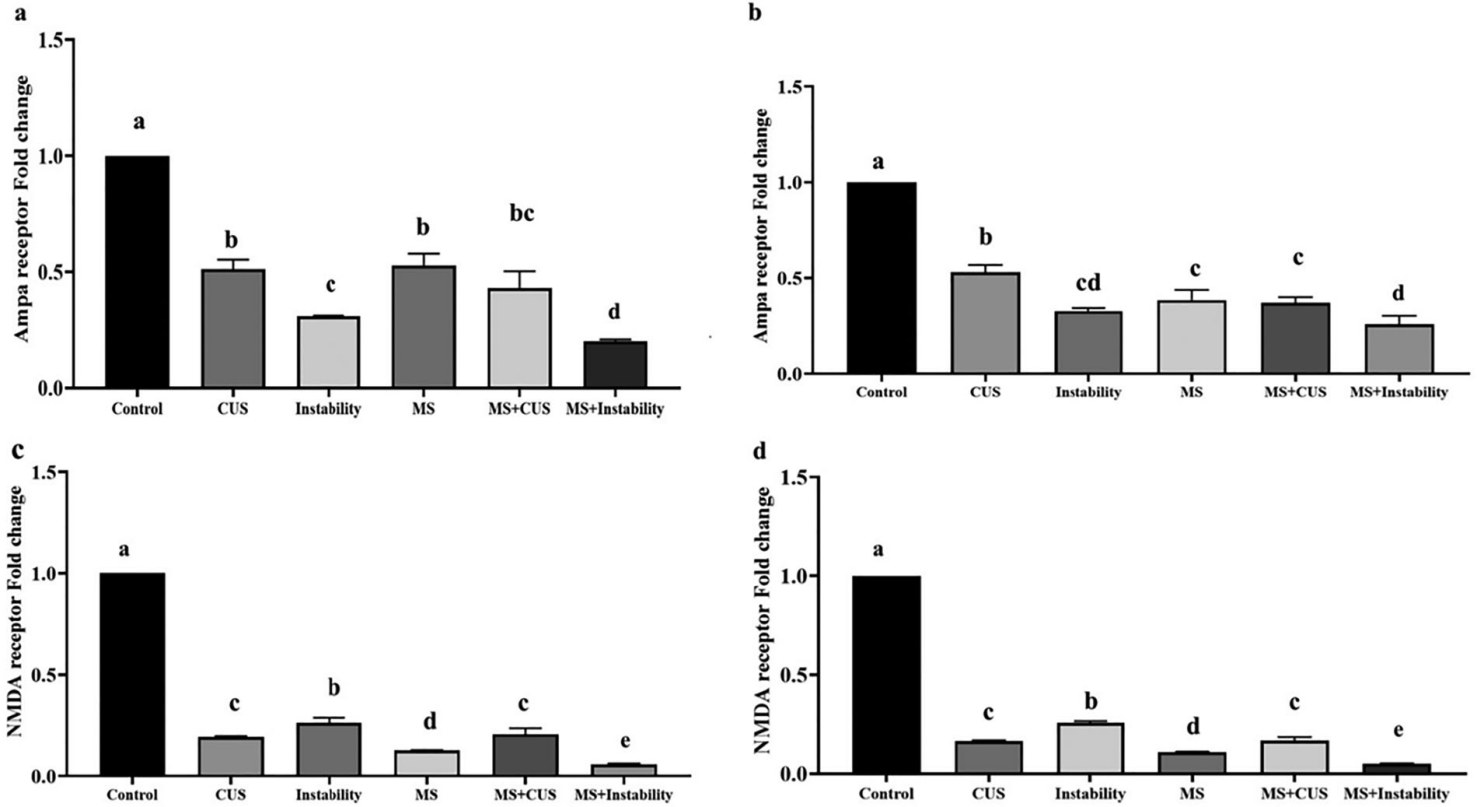
Illustrates the impact of various stressors on the gene expression of AMPA and NMDA in the hippocampal tissue (a and c) and the prefrontal cortex (b and d). Each point represents the mean ± SEM. Maternal stress (MS) group, social instability stress (Instability) group, and chronic unpredictable stress (CUS) group. Groups that differ significantly are indicated with a different letter (*p* < 0.05).

Statistical analysis showed that there was a significant decrease in the gene expression of AMPA and NMDA receptor mRNA levels in the prefrontal and hippocampal tissues of the MS, CUS, and instability groups. This decrease was about 2.5 times compared to the control group. Additionally, in the CUS + MS and instability + MS groups, where maternal stress was present, there was a decrease in the expression of AMPA and NMDA genes, surpassing the levels observed in the MS group. Importantly, the expression level in the CUS + MS and instability + MS groups showed a significant decrease compared to the maternal stress group (Figure 6).

## 4 Discussion

The fetal period represents a critical stage in human development, exerting a significant influence on both growth and mental health outcomes. Prenatal stress can lead to enduring effects on mental health and overall human development throughout an individual's lifespan. This study examined the long‐term consequences of prenatal stress on cognitive abilities and related gene expression in specific brain regions. To this end, female rats were subjected to restraint stress during pregnancy, and their male offspring were later exposed to social instability stress and CUS during adulthood. Through a series of behavioral tests and an analysis of gene expression in the hippocampus and frontal cortex, the study found that the rats that experienced prenatal stress exhibited persistent deficits in these cognitive functions. Furthermore, these impairments worsened when the rats were subjected to chronic stress in adulthood. Also, we found the altered patterns of gene expression, including decreased mRNA levels of BDNF and TrkB, as well as NMDA and AMPA receptors, coupled with increased TLR2/TLR4 gene expression. This can indicate enduring deficits in memory formation and retrieval due to prenatal stress, which are further exacerbated by chronic stressors in adulthood. Additionally, these findings demonstrate the complex interplay between prenatal stress, gene expression, and cognitive outcomes.

Our results showed that rats in the MS + CUS and MS + Instability groups showed significantly longer latencies and traveled greater distances to find the hidden platform during the acquisition phase of the MWM test. While acquisition trials in the passive avoidance test revealed no significant differences among groups, the STL during retention trials was significantly reduced in the MS + CUS and MS + Instability groups. The probe test further confirmed these deficits, as stressed animals spent less time and covered shorter distances in the target quadrant, indicating reduced memory retention.

In line with our findings, several studies conducted on humans and animal models have shown the detrimental effects of stress during pregnancy on memory and learning from the molecular level to the behavioral level (Li et al. 2020; Lopatina et al. 2021). According to studies, the mother's exposure to stress during pregnancy strengthens the adverse effect of acute stress on the synaptic plasticity of hippocampal neurons and also destroys memory and spatial learning (Lopatina et al. 2021). Experiencing stress during gestation increases the risk of mood disorders in the offspring. It has been reported that the adult offspring of rodents exposed to maternal stress exhibited depressive‐like phenotypes (Amani et al. 2021). In addition, they were more susceptible to stress and anxiety (Boersma and Tamashiro 2015). During the development of the brain and throughout life, the prenatal environment may influence the behavioral phenotype of an adult. The vulnerability of the brain to stressors during the perinatal period can be attributed to the high level of plasticity in the brain at this specific time (Kundakovic and Jaric 2017).

These findings are consistent with previous studies demonstrating that chronic stress hampers hippocampal‐dependent tasks and alters dendritic morphology in CA3 pyramidal neurons (Suri and Vaidya 2015). Additionally, early‐life stress has been shown to prime the hypothalamic‐pituitary‐adrenal (HPA) axis for heightened responsiveness, thereby exacerbating the neurobiological effects of later stress exposure (Huang 2014). The reduced STL in the compounded stress groups supports the hypothesis that cumulative stress disrupts long‐term memory consolidation, potentially through alterations in glucocorticoid receptor expression and impaired synaptic plasticity (Frumento 2022). These findings resonate with previous research showing that early‐life adversity can result in enduring changes in brain structure and increased cognitive vulnerability (Saleh et al. 2017).

Based on our results, the NMDA and AMPA receptors decreased following stress. The significant downregulation of AMPA and NMDA receptor mRNA in stressed animals indicates compromised excitatory neurotransmission. These ionotropic glutamate receptors are fundamental to synaptic efficacy and are heavily involved in memory encoding and retrieval (Mukherjee and Manahan‐Vaughan 2013). Stress‐induced reductions in their expression may impair long‐term potentiation and synaptic remodeling, contributing to the observed deficits in both spatial and aversive memory tasks. Chronic stress–induced downregulation of ionotropic glutamate receptors in both the hippocampus and prefrontal cortex has been widely reported. For instance, CUS selectively decreases AMPAR‐mediated synaptic excitation at hippocampal temporoammonic‐CA1 synapses and reduces GluA1 expression in that pathway (Kallarackal et al. 2013). Acute stress similarly causes a marked, subunit‐specific drop in Ser831‐GluA1 phosphorylation in the medial hippocampal and prefrontal cortex and dorsal hippocampus, reflecting diminished AMPAR function in these regions (Caudal et al. 2010). Consistent with the current findings, previous studies have shown that chronic stress can alter glutamate receptor trafficking and reduce receptor density in the hippocampus and prefrontal cortex (Lee et al. 2022). Moreover, glucocorticoid‐driven mechanisms have been shown to lower both NMDA and AMPA receptor expression in hippocampal and prefrontal neurons, linking HPA‐axis activation to impaired glutamatergic signaling under stress (Gulyaeva 2021). The stress‐induced downregulation of NMDA and AMPA receptors in the hippocampus and prefrontal cortex points to a fundamental weakening of excitatory synaptic transmission and plasticity, mechanisms that directly underlie the learning and memory impairments observed in our study (Kallarackal et al. 2013).

Our findings show that experiencing stress significantly reduced BDNF expression in the brain. These results align with a growing number of studies indicating that chronic stress leads to downregulation of BDNF in the hippocampus and prefrontal cortex (Samandari‐Bahraseman et al. 2024). For example, maternal separation and unpredictable mild stress both decrease long 3′UTR Bdnf mRNA in these regions, impairing synaptic plasticity and cognitive performance (Robinson et al. 2021)(Robinson et al. 2021). At the epigenetic level, stress‐induced enrichment of H3K9me2 at the BDNF locus in the hippocampus and medial hippocampal and prefrontal cortex has been shown to repress BDNF transcription (Jiang et al. 2021). Moreover, chronic restraint stress upregulates the translational suppressor Pdcd4 in the hippocampus, leading to marked reductions in BDNF protein and attendant behavioral deficits (Li et al. 2021). BDNF plays a critical role in neuronal development by regulating the survival, differentiation, and maintenance of function across diverse neuronal populations (Gonzalez et al. 2016). Considering the effect of this essential neurotrophic factor on survival, synaptic plasticity and release of neurotransmitters, it seems that the reduction of its expression can induce detrimental effects on many mental and behavioral activities (Mohammadinia, F etal.(2024)Autry and Monteggia 2012). BDNF is crucial for neuronal development and may influence neuronal signaling in stressed dams during gestation (Notaras and van den Buuse 2020; Yu and Chen 2011). Decreased expression of BDNF may decrease synaptic plasticity and dendritic arborization (Kowiański et al. 2018). Based on the literature, prenatal stress affects the connectivity between the amygdala and the prefrontal cortex, which matures during early adolescence. Disruptions in this connectivity have been linked to depression and emotional issues (Park et al. 2018; Yau et al. 2011).

We found a decrease in TrkB mRNA in stress‐experiencing rats, which parallels findings from similar studies. Fang et al. (2020) showed that 4 weeks of chronic unpredictable mild stress reduces TrkB transcript levels in CA1 (Fang et al. 2020), the dentate gyrus, and the medial hippocampal and prefrontal cortex, paralleling the onset of depressive‐like behaviors and spatial memory deficits (Ray et al. 2011). Also, it has been reported that 21 days of restraint stress markedly lowers hippocampal TrkB mRNA, impairs long‐term potentiation at Schaffer collateral–CA1 synapses, and slows acquisition in the Morris water maze (Berghauzen‐Maciejewska et al. 2015). Similarly, Fang et al. (2021) found that repeated social defeat stress decreases TrkB expression in the medial hippocampal and prefrontal cortex and correlates with working‐memory impairments in a Y‐maze task (Fang et al. 2020). TrkB is a high‐affinity receptor tyrosine kinase for neurotrophins, principally BDNF, mediating their trophic effects on neurons and glia. Taken together, it can be suggested that diverse chronic stressors converge on TrkB suppression, undermining neurotrophic support and synaptic plasticity as a common pathway for stress‐induced cognitive dysfunction.

Elevated expression of TLR2 and TLR4 in stressed groups, particularly those exposed to both maternal and adult stress, suggests a pronounced neuroinflammatory response. Toll‐like receptors are key mediators of innate immunity and have been implicated in stress‐induced activation of microglia and release of pro‐inflammatory cytokines (Nie et al. 2018).

Our finding of stress‐induced upregulation of TLR2 and TLR4 in both the hippocampus and prefrontal cortex also mirrors several prior reports showing that chronic stress engages innate immune receptors in the brain. For instance, Souza‐Junior et al. (2022) demonstrated that repeated stress elevates TLR4 expression in hippocampal and prefrontal cortex microglia, driving neuroinflammation and anxiety‐like behavior (Souza‐Junior et al. 2022). Likewise, Amiresmaeili et al. (2018) found that CUS significantly increases both TLR2 and TLR4 mRNA levels in the rat hippocampus, correlating with depressive‐like phenotypes (Amiresmaeili et al. 2018). Similarly, a recent comprehensive review further emphasizes that stress‐triggered TLR2/4 activation initiates pro‐inflammatory signaling cascades that impair synaptic plasticity and contribute to cognitive and mood disturbances (Abarca‐Merlin et al. 2024). Following chronic stress and continuous contact with glucocorticoids, several signalling pathways, including TLRs, are affected in the occurrence of depression symptoms and other psychological disorders (Troubat et al. 2021; Welcome and Mastorakis 2020). In particular, TLR2 and TLR4 have been identified as critical mediators of neuroinflammation, which leads to neuronal changes and anxiety (Nie et al. 2018; Pascual et al. 2015).

## 5 Conclusion

Our study demonstrates that prenatal restraint stress is associated with impairments in learning and memory, evident across passive and active avoidance tasks as well as spatial navigation tasks, which are further exacerbated by chronic unpredictable stress and social instability in adulthood. At a molecular level, we show that early‐life adversity downregulates hippocampal and prefrontal expression of key neurotrophic and excitatory receptors (BDNF, TrkB, NMDA, AMPA) while upregulating innate immune sensors (TLR2/4), highlighting a long‐lasting reprogramming of neurotrophic and neuroimmune pathways. These findings underscore how prenatal stress primes the brain for heightened vulnerability to later‐life challenges, offering potential targets for intervention.

Nonetheless, our work has several limitations: only male offspring were examined, preventing assessment of sex‐dependent effects; gene expression analyses were confined to mRNA levels without protein or functional validation; and investigations were restricted to two brain regions and select molecular markers, leaving broader circuit‐level and systemic impacts unaddressed. Future studies should include both sexes, longitudinal and proteomic approaches, electrophysiological assays, and interventional paradigms to fully elucidate mechanisms and therapeutic strategies for stress‐induced cognitive dysfunction.

## Author Contributions

**Manijeh Dogani**: data curation, investigation, formal analysis, writing – original draft, and editing. **Nayere Askari**: project administration, formal analysis, investigation writing – original draft, and editing. **Mohammad Reza Vaez‐Mahdavi**: supervision, writing – original draft, and editing.

## Funding

The authors have nothing to report.

## Conflicts of Interest

The authors declare no conflicts of interest

## Data Availability

The complete datasets generated during the current study are available from the corresponding author on request.
